# Reply to: Comment on ‘Molecular evidence of viral DNA in non-small cell lung cancer and non-neoplastic lung’

**DOI:** 10.1038/bjc.2016.339

**Published:** 2016-10-20

**Authors:** Lary A Robinson, Crystal J Jaing, Christine Pierce Campbell, Anthony Magliocco, Yin Xiong, Genevra Magliocco, James B Thissen, Scott Antonia

**Affiliations:** 1 Department of Thoracic Oncology, Moffitt Cancer Center, Tampa, FL 33612-9416, USA; 2 Center for Infection Research in Cancer (CIRC), Moffitt Cancer Center, Tampa, FL 33612-9416, USA; 3 Physical and Life Sciences Directorate, Lawrence Livermore National Laboratory, Livermore, CA 94559-9698, USA; 4 Department of Epidemiology, Moffitt Cancer Center, Tampa, FL 33612-9416, USA; 5 Department of Pathology, Moffitt Cancer Center, Tampa, FL 33612-9416, USA

**Sir,**

After reviewing the published literature that documented an estimated 20% of newly diagnosed cancer cases worldwide are caused by infectious agents (Sarid and Shou-Jiang, 2011; zur Hausen, 2011), and the numerous studies that suggested a viral link (primarily human papillomavirus (HPV)) to non-small cell lung cancer (NSCLC; Klein *et al*, 2009), we chose to forego the standard narrowly focused PCR technique in favour of a generalised, non-targeted approach to detect microbial DNA/RNA in frozen, archived NSCLC specimens. Therefore, in our discovery study to screen for potential microorganisms in NSCLC (Robinson *et al*, 2016), we employed the novel Lawrence Livermore Microbial Detection Array (LLMDA) designed to detect all sequenced viral and bacterial families, with appropriate controls that target all vertebrate pathogens, including 1856 viruses, 1398 bacteria, 125 archaea, 48 fungi, and 94 protozoa (Gardner *et al*, 2010; Victoria *et al*, 2010; Erlandsson *et al*, 2011). Microarrays span the middle ground between PCR and sequencing, offering high probe density to detect diverse targets with lower costs and fast turnaround time.

As expected, we found that a large number of squamous cell carcinomas contained DNA from HPV, of which 30% were high-risk HPV. Surprisingly, exogenous retroviruses of several varieties were found in 85% of squamous cell carcinomas and 47% of adenocarcinomas. Retroviruses are known oncoviruses in numerous non-human animal cancers (including adenocarcinoma of the lung) and in at least one human cancer (adult T-cell leukaemia). However, a most intriguing unexpected finding was the high incidence of DNA from hepatitis B virus (HBV) in 9/13 (69%) of squamous cell carcinomas and 12% of adenocarcinomas. HBV is a known oncovirus causing liver cancer (Di Bisceglie, 2011) and may be involved in multiple myeloma (Li *et al*, 2015), but its relationship to lung malignancies is unclear.

In this Letter to the Editor, the authors present a succinct and convincing discussion describing how HBV might play a role in squamous cell carcinoma of the lung, as it relates to inflammation and hit-and-run carcinogenesis. However, we emphasise caution – the detection of viral DNA (HBV, retroviruses, or HPV) in tumour tissue does not imply causation. Nevertheless, we now appear to have a wider field of suspect microorganisms to investigate in future studies of lung cancer. Our research group is currently employing numerous techniques to substantiate our initial findings and evaluate whether a microbial signature may identify individuals at high risk for this prevalent and lethal cancer.
